# YTHDF2, a protein repressed by miR-145, regulates proliferation, apoptosis, and migration in ovarian cancer cells

**DOI:** 10.1186/s13048-020-00717-5

**Published:** 2020-09-18

**Authors:** Jie Li, Lei Wu, Meili Pei, Yun Zhang

**Affiliations:** 1 Department of Pathology, the First Affiliated Hospital of Xi’an Jiaotong University, 277 West Yanta Road, Xi’an, 710061 Shaanxi China; 2 Department of Gynecology and Obstetrics, the First Affiliated Hospital of Xi’an Jiaotong University, Xi’an, China

**Keywords:** YTHDF2, m6A, miR-145, Ovarian cancer

## Abstract

RNA methylation can reverse the methylation modification at the RNA level, which is an extremely important epigenetic modification. The function and mechanism of YTHDF2, as a reader of m6A modification, in epithelial ovarian cancer (EOC) have not been elucidated so far. This study aimed to investigate how YTHDF2 and miR-145 modulated EOC progression through m6A modification. It demonstrated that YTHDF2 was significantly upregulated in EOC tissues compared with normal ovarian tissues. Further functional studies confirmed that YTHDF2 significantly promoted the proliferation and migration of EOC cell lines and reduced the global 6-methyladenine (m6A) mRNA levels. Next, the expression levels of miR-145 and YTHDF2 were found to be inversely correlated in ovarian cancer tissues and cells, and YTHDF2 was the direct target gene of miR-145. A crucial crosstalk occurred between miR-145 and YTHDF2 via a double-negative feedback loop. The overexpression of YTHDF2 rescued miR-145-induced reduction of the proliferation and migration of EOC cells. Hence, YTHDF2 and miR-145, as two crucial m6A regulators, were involved in the progression of EOC by indirectly modulating m6A levels. The findings of this study on YTHDF2 and miR-145 might provide new insights into carcinogenesis and new potential therapeutic targets for EOC.

## Introduction

Ovarian cancer is a common malignant tumor of the female reproductive system, and its mortality rate ranks first among gynecological tumors [1]. It has many histopathological types, about 90% of which belong to epithelial ovarian cancer (EOC). Patients with EOC have no obvious symptoms and signs in the early stage and lack effective means of early screening. Consequently, most of them reach the late stage before a clear diagnosis. The main treatment of EOC is surgery, supplemented by intravenous chemotherapy or intraperitoneal perfusion chemotherapy or combination therapy. However, the treatment has not achieved satisfactory results; the 5-year survival rate of patients with EOC is still around 40% [2]. Besides the difficulty in early diagnosis, the poor prognosis of patients is also attributed to tumor recurrence and metastasis [3]. Therefore, early sensitive markers of EOC should be discovered, key molecules related to EOC recurrence and metastasis should be identified, and a targeted and multi-functional gene therapy should be developed on an urgent basis.

RNA methylation can reverse the methylation modification at the RNA level, which is an extremely important epigenetic modification [4]. Further, 6-methyladenine (m6A) is the most abundant methylation modification of mRNA in eukaryotic cells, involving the participation of three types of molecules: methyltransferase complex (METTL3, METTL14, and WTAP), named as “Writers”; demethylase (FTO and ALKBH5), named as “Erasers”; and m6A-modified binding protein (YTHDF1/2/3), named as “readers”, which can dynamically and reversibly regulate the m6A level [5, 6]. In 2017, Professor Chuan’s research team found that m6A was involved in the regulation of self-renewal and differentiation of glioblastoma stem cells (GSCs). In vitro and in vivo experiments showed that the knockout of FOXM1-AS and ALKBH5 affected the tumorigenicity of GSCs related to FOXM1, revealing the importance of demethylase ALKBH5 and m6A in glioblastoma [7]. Shuhan et al. revealed the significance of METTL14 in inhibiting the metastasis of HCC [8]. Jaffrey et al. found that METLL3 regulated the myeloid differentiation of normal hematopoietic stem cells and leukemic cells, thus providing more basis for METTL3 as a potential target for myeloid leukemia treatment [9].

Readers are responsible for “reading” the information of RNA methylation modification and participating in the process of downstream RNA translation and degradation. “Reading” has two modes. One is direct reading, which refers to the selective binding with the m6A site of RNA. The earliest readers belong to the YTH domain family of proteins, including YTHDF and YTHDC subtypes, such as YTHDC1, YTHDC2, YTHDF1, YTHDF2, and YTHDF3 [10]. YTHDF2 can accelerate the decay of m6A methylated mRNA [11, 12]. YTHDF2 was found to regulate m6A levels in HCC [13]. However, the expression and mechanism of YTHDF2 in most tumors, especially in ovarian cancer, have not been elucidated so far.

MicroRNAs (miRNAs) are a kind of endogenous noncoding microRNAs with a length of about 22 nucleotides, which widely exist in eukaryotes. A correlation was observed between the methylation modification of RNA m6A and miRNA. On the one hand, the miRNA-targeting site showed that m6A was enriched, and miRNA could positively regulate the activity of METTL3; on the other hand, the miRNA synthesis depended on m6A methylation modification [14, 15]. The expression of miR-145 was significantly lower in breast cancer, cervical cancer, glioma, colon cancer, esophageal cancer, and nonsmall cell lung cancer [16]. MiR-145 has different regulatory genes in different tumors; the molecular mechanisms of tumor suppressors are also different. Previous studies confirmed that miR-145 could regulate different biological functions of ovarian cancer by targeting different target genes [17–20]. However, the expression pattern and the m6A-regulated role of miR-145 in ovarian cancer were still unclear.

The present study found that YTHDF2 and miR-145 formed a negative feedback pathway to regulate ovarian cancer progression through m6A modification. The results provided a theoretical basis for the application of YTHDF2 and miR-145 in the diagnosis and treatment of ovarian cancer.

## Materails and methods

### Human tissue specimens and cell culture

SKOV3 was obtained from the Shanghai Cell Bank of Chinese Academy of Sciences (Shanghai, China), 3AO was from the Shandong Academy of Medical Sciences (Jinan, China). Cells were maintained in RPMI 1640 medium (Gibco-BRL, Gaithersburg, MD, USA) supplemented with 10% (v/v) fetal bovine serum at 37 °C under a humidified 5% CO_2_ atmosphere. Human ovarian cancer tissue samples and normal ovarian tissue samples were collected from patients at The First Affiliated Hospital of Xi’an Jiaotong University, PR China. This study was approved by the Ethics Committee of The First Affiliated Hospital of Xi’an Jiaotong University, China.

### Plasmid transfection

The human YTHDF2 expression vector pcDNA3-flag-YTHDF2 were obtained from Addgene (Boston, MA, USA). Cells were seeded into 6-well plates until 70–90% confluency and transiently transfected with pcDNA3-flag-YTHDF2 or empty vector using the X-treme GENE HP DNA Transfection Reagent (Roche, Indianapolis, IN, USA) following the manufacturer’s protocol.

### siRNA and transient transfection

Human YTHDF2 siRNA were purchased from GenePharma (Shanghai, China). YTHDF2 siRNA was transiently transfected 100 nM per well using the X-treme GENE siRNA Transfection Reagent (Roche, Indianapolis, IN, USA) following the manufacturer’s protocol. RNA was extracted 48 h later and protein was extracted 72 h later for subsequent experiments.

### miR transient transfection

miR-145 mimic and negative control were purchased from Ribo-Bio Co. Ltd. (Guangzhou, China). SKOV3 and 3AO cells were transiently transfected with 60 nM miR-145 mimic or negative control using the X-treme GENE siRNA Transfection Reagent (Roche, Indianapolis, IN, USA) following the manufacturer’s protocol.

### Quantitative real-time PCR (qRT-PCR)

Total RNA was extracted from cells using TRIzol reagent (Invitrogen, Carlsbad, CA, USA) according to the manufacturer’s instructions. For mRNA detection, first-strand cDNA was synthesized using a RevertAid first strand cDNA synthesis Kit (Thermo Fisher Scientific Inc., Waltham, MA, USA). Quantitative real-time PCR was performed using a SYBR Premix Ex Taq™ II kit (Takara, Dalian, China) on a CFX96 real-time PCR system (Bio- Rad, Hercules, CA, USA).

### Western blot

Total proteins were extracted by RIPA lysis buffer (Roche, Indianapolis, IN, USA) and 1 mM PMSF on ice, proteins were separated by SDS-PAGE and then transmembrane. 5% skimmed milk was sealed at room temperature for 2 h, and then incubated overnight at 4 °C with rabbit anti-human YTHDF2(1:1000, Cell Signaling Technology, Danvers, MA, USA). TBST membrane was washed for 8 min and 5 times, and the corresponding second antibody (1:2000) was added, incubated for 2 h, and TBST membrane was washed for 8 min and 5 times.

### Luciferase reporter assay

Cells were co-transfected with pRL-TK vector (20 ng), wild-type (WT-3′ UTR) or mutant (MUT-3′ UTR) reporter vectors (180 ng), along with miR-145 mimic or negative control at a final concentration of 20 nM using the X-treme GENE siRNA Transfection Reagent. 24 h after transfection, the relative firefly luciferase activity (normalized to Renilla luciferase activity) was measured using a dual-luciferase reporter gene assay system (Promega, Madison, WI, USA), and results were depicted as the percentage change over the respective control.

### RNA m6A quantitative experiment

In this experiment, the total RNA content of m6A was determined by using the m6A RNA metrology Quantification Kit (ab185912, Abcam) of Abcam company. We measured m6A level following the manufacturer’s protocol. The absorbance of the measuring plate at 450 nm was measured by the enzyme scale instrument, and the RNA m6A content of each sample was calculated according to the standard curve. The formula is m6A% = [(sample OD-NC OD)/S] / [(PC OD-NC OD)/P] × 100%, where S is the ng amount of sample RNA and P is the ng amount of positive control RNA.

### Cell viability assay

Cells in logarithmic growth phase were inoculated into 96-well plates with 5000 holes per hole, 100 μL of culture medium was added into each hole and incubated overnight in a 37 °C, 5% CO_2_ incubator, then add berberine for 48 h. 10 μL CCK8(7Sea, Shanghai, China) was added to each pore and incubated at 37 °C for 4 h. The absorbance value of each pore OD 450 was determined by enzyme labeling (PerkinElmer, Waltham, MA, USA).

### Transwell assay

Cells were trypsinized and counted. A total of 1 × 10^5^ cells in 100 μl serum-free medium were added into millicells (Millipore Co., Bedford, MA, USA). 500 μl of 1640 medium containing 20% newborn bovine serum was added to the bottom chambers as the chemotactic factor. After incubation for 24 at 37 °C. Migratory cells were counted and averaged from images of five random fields (original magnification × 200) captured using an inverted light microscope. Each cell count was performed by three researchers.

### Cell apoptosis assay

Cell apoptosis analysis was performed using an Annexin V-FITC/propidium iodide (PI) Apoptosis Detection kit (KeyGEN Biotech, Nanjing, China). Normal culture cells were selected in the logarithmic growth period, and the growth state was good for the experiment. After culture for 24 h, the supernatant was introduced into EP tube, and cells were digested with trypsin without EDTA, then cell suspension was made and transferred to new EP tube. After centrifuging for 10 min at 1000 rpm and 4 °C, discard the supernatant; add 1 ml of precooled PBS, gently blow to suspend the cells for 1000 rpm, centrifuging for 10 min at 4 °C, discard the supernatant; repeat step 3 and step 4 twice; re suspend the cells in 400 μl × binding buffer; add 5 μl annexin V-FITC to each sample to be tested, and add PI after mixing 5 μl, mix well, react at room temperature for 15 min, pay attention to avoid light, and try to get on the machine within 1 h. The results were analyzed using the Cell-Quest™ Pro software (BD Biosciences, Bedford, MA, USA).

### Statistical analysis

Data were presented as the means±SE and were analyzed using SPSS 22.0 software (Chicago, IL, USA). Statistical differences were tested by Chi-square test, two-tailed t-test, one-way ANOVA test or Fisher’s Exact test. Differences were considered significant at *P* < 0.05(*) or highly significant at *P* < 0.001 (**).

## Results

### Expression of YTHDF2 in ovarian cancer tissues

**Fig. 1:**
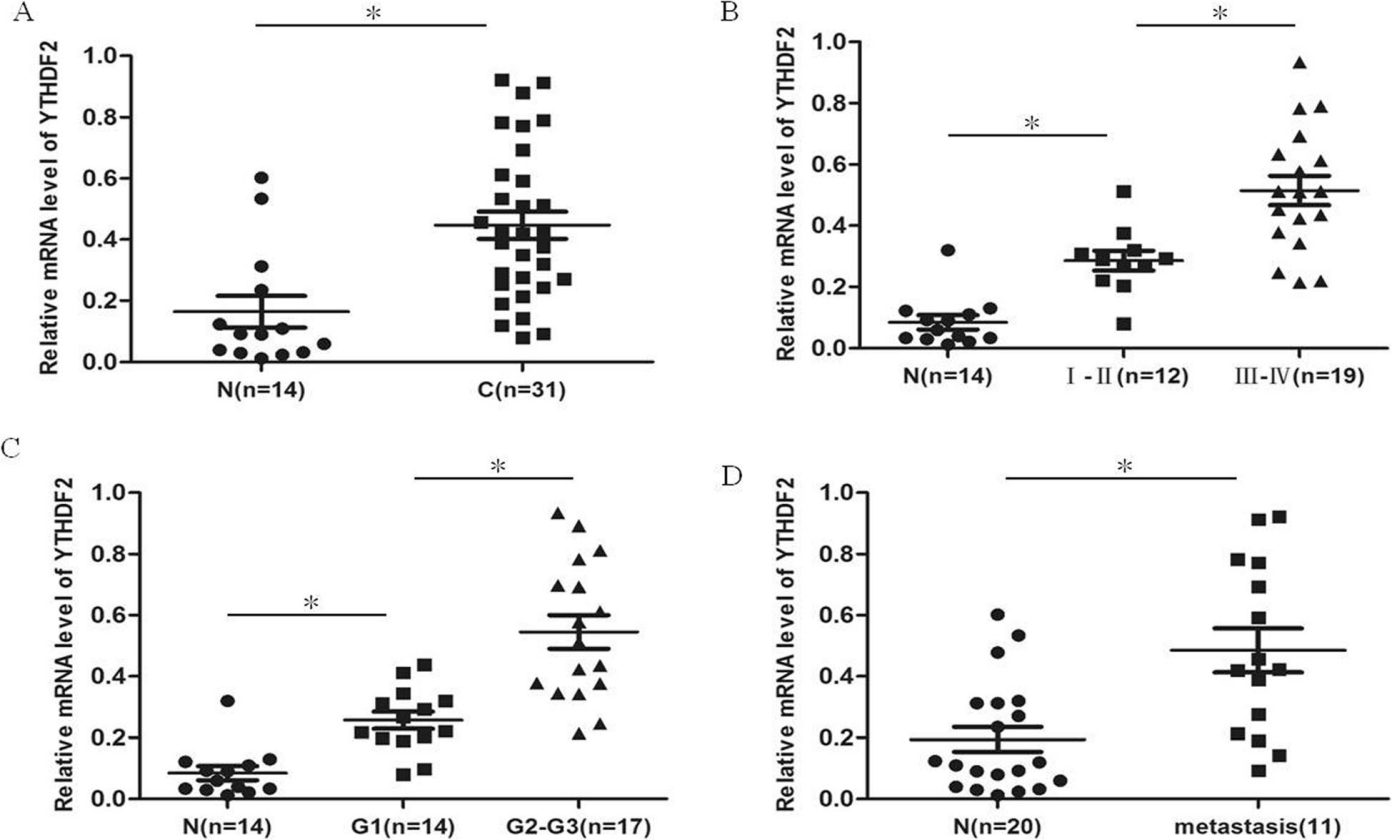
The expression of YTHDF2 in ovarian cancer tissues. **a** Relative expression of YTHDF2 in ovarian cancer tissues and normal ovarian tissues. **b** The relationship between the expression level of YTHDF2 and clinical stage. **c** The relationship between the expression level of YTHDF2 and pathological grade. **d** The relationship between the expression level of YTHDF2 and metastasis. All experiments were carried out in triplicate and the results were presented as means ± SE. *P < 0.05, **P < 0.001, t-test. N normal ovarian tissue, C ovarian cancer tissues

An increasing number of studies have shown that m6A modification is vital in the occurrence and development of complex human diseases, especially cancer. However, its specific expression patterns in EOC are still unclear. The present study reported the mRNA expression of YTHDF2 in ovarian cancer tissues and normal ovarian tissues. The expression of YTHDF2 was higher in ovarian cancer tissues than in normal ovarian tissues (Fig. 1a). The clinicopathological correlation analysis of the DNMT3A level in ovarian cancer showed that the later the clinical stage, the higher the pathological grade, the higher the expression of YTHDF2, and the higher the expression of YTHDF2 in patients with metastasis (Fig. 1b–d). The results indicated that YTHDF2 promoted ovarian cancer progression.

### YTHDF2 significantly downregulated global mRNA m6A expression to promote EOC cell proliferation and migration

**Fig. 2:**
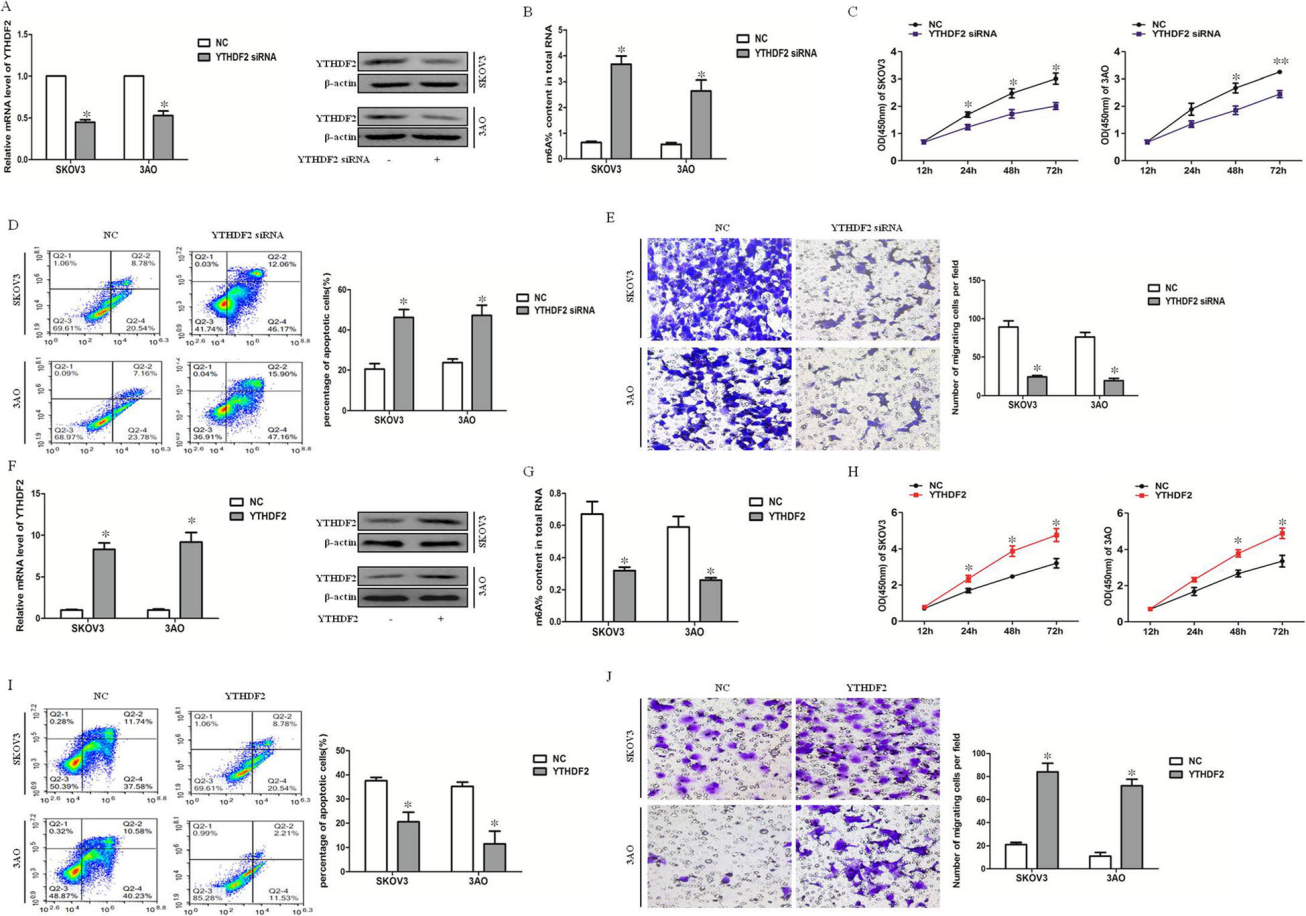
YTHDF2 significantly downregulates global mRNA m6A levels to promotes EOC cell proliferation and migration. **a** The mRNA and protein level of YTHDF2 was downregulated after transfection of YTHDF2 siRNA. **b** Knocking down YTHDF2 increased global mRNA m6A level. (C)CCK8 assay result showed knock-down of YTHDF2 decreased proliferation of ovarian cancer cells. **d** Knock-down of YTHDF2 promoted apoptosis of ovarian cancer cells. **e** Knock-down of YTHDF2 inhibited migration of ovarian cancer cells. **f** The mRNA and protein level of YTHDF2 was upregulated after overexpression of YTHDF2. **g** overexpression of YTHDF2 decreased global mRNA m6A level. (H)CCK8 assay result showed upregulated of YTHDF2 increased proliferation of ovarian cancer cells. (I) overexpression of YTHDF2 inhibited apoptosis of ovarian cancer cells. **j** overexpression of YTHDF2 promoted migration of ovarian cancer cells. All experiments were carried out in triplicate and the results were presented as means ± SE. *P < 0.05, **P < 0.001, t-test

An RNA m6A quantitative experiment was performed to investigate global mRNA m6A expresssion. After the downregulation of YTHDF2 (Fig. 2a), the global mRNA m6A expression was upregulated (Fig. 2b). The CCK8 test results showed that the proliferation of ovarian cancer cells decreased after knocking down YTHDF2 (Fig. 2c). Accordingly, cell apoptosis increased (Fig. 2d) and migration decreased (Fig. 2e). Next, after the overexpression of YTHDF2 (Fig. 2f), the global mRNA m6A level decreased (Fig. 2g), the proliferation of ovarian cancer cells increased (Fig. 2h), cell apoptosis decreased (Fig. 2i), and migration increased (Fig. 2j). In conclusion, YTHDF2, as an important m6A reader, significantly promoted proliferation and migration by decreasing the global mRNA m6A levels in ovarian cancer cells, suggesting the involvement of m6A modification and the reader protein YTHDF2 in the carcinogenesis of EOC.

### YTHDF2 was the direct target gene of miR-145

**Fig. 3:**
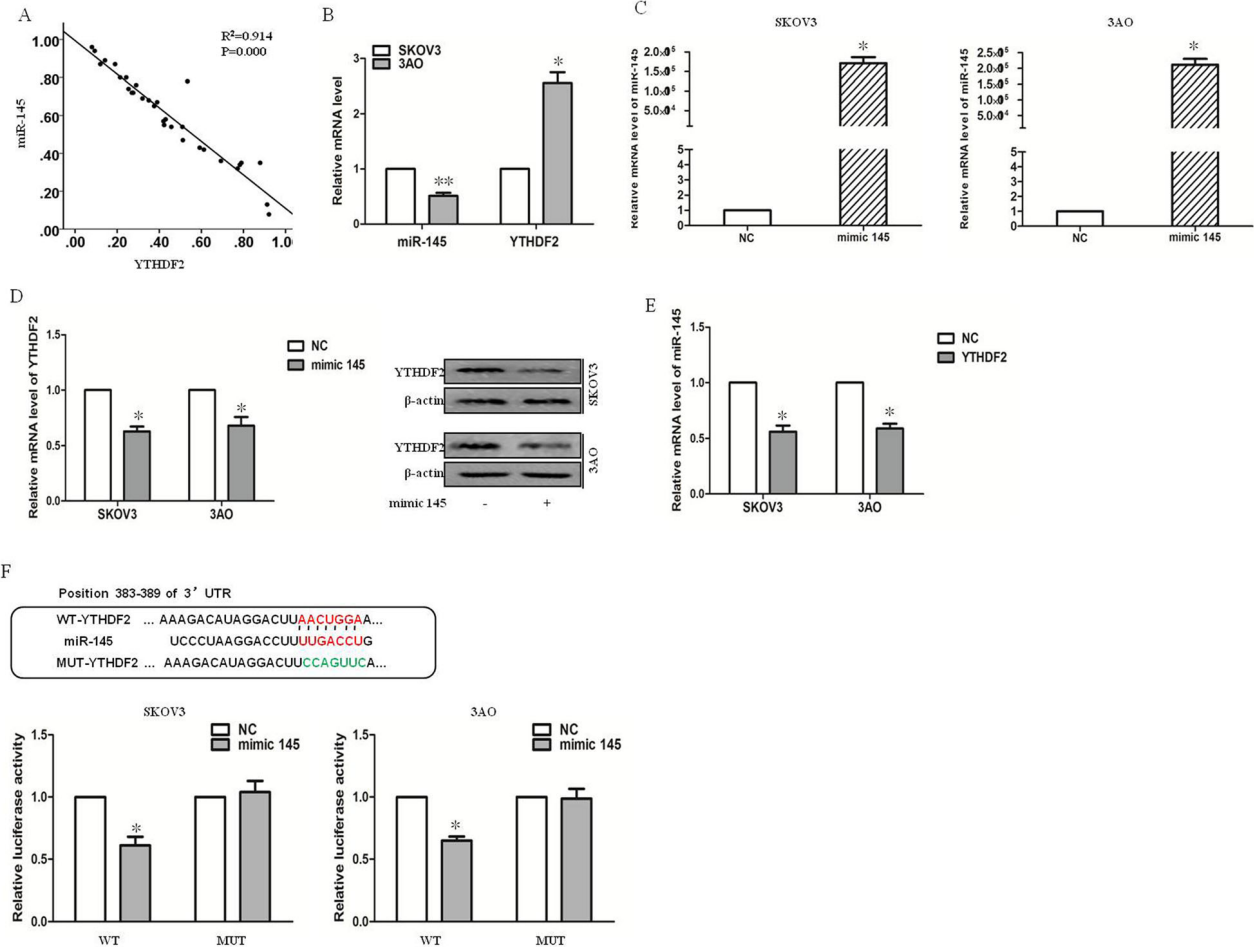
YTHDF2 is the direct target gene of miR-145. **a** Scatter diagram showing. YTHDF2 expression and miR-145 expression by qRT-PCR and their correlations (R^2^ = 0.914, *P* = 0.000) in 31 EOC tissue samples. **b** qRT-PCR results showed a negative correlation between YTHDF2 and miR-145 in both SKOV3 and 3AO cells. **c** qRT-PCR showed that transfection of miR-145 mimic rescued miR-145 level in SKOV3 and 3AO cells. **d** The mRNA and protein levels of YTHDF2 decreased after overexpression of miR-145. **e** The expression level of miR-145 increased after overexpression of YTHDF2. **f** Luciferase reporter assays showed that miR-145 targeted YTHDF2 directly. All experiments were carried out in triplicate and the results were presented as means ± SE. *P < 0.05, **P < 0.001, t-test

A previous study demonstrated low expression of miR-145 in ovarian cancer. The present study further analyzed the mRNA expression levels of miR-145 and YTHDF2 in ovarian cancer tissues. The results showed that the expression levels of miR-145 and YTHDF2 had an inverse correlation in ovarian cancer (Fig. 3a). The YTHDF2 level was higher in the miR-145 low-expression cell line than in the miR-145 high-expression cell line (Fig. 3b). After the overexpression of miR-145 (Fig. 3c), the mRNA and protein expression levels of YTHDF2 decreased (Fig. 3d). The expression level of miR-145 also decreased (Fig. 3e) after the overexpression of YTHDF2 (Fig. 2f), suggesting a crucial crosstalk between miR-145 and YTHDF2 via a double-negative feedback loop. TargetScan (http:// www.targetscan.org/, http://www.mirdb.org/) predicted that YTHDF2 was a target gene of miR-145. The luciferase reporter assay revealed that miR-145 targeted YTHDF2 directly (Fig. 3f). In a word, YTHDF2 was the direct target gene of miR-145.

### Overexpression of YTHDF2 rescued miR-145-induced reduction of the proliferation and migration of EOC cells

**Fig. 4:**
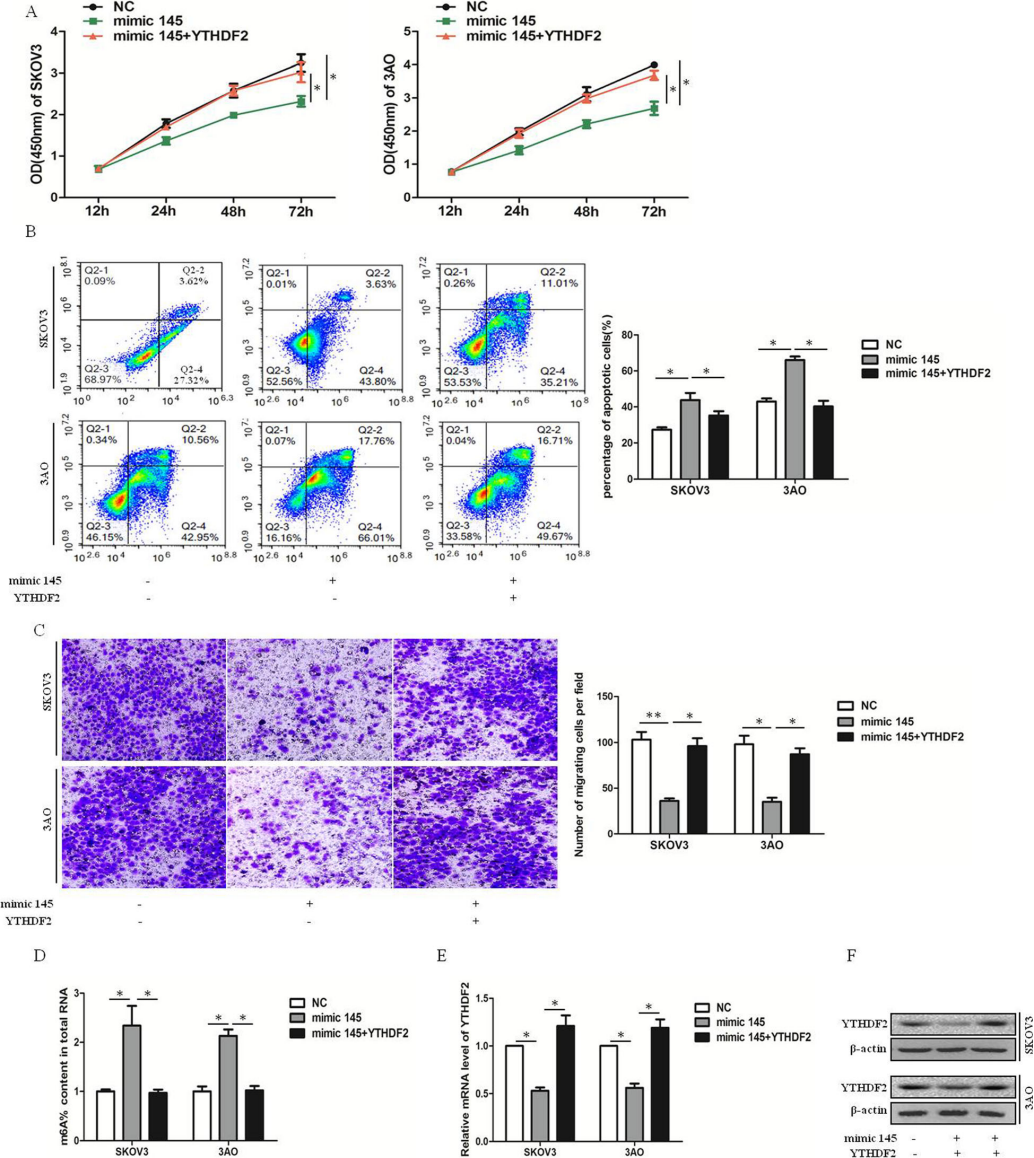
Overexpression of YTHDF2 rescues miR-145-induced reduction of proliferation and migration in EOC. **a** overexpression of YTHDF2 reversed the proliferation repressed by overexpression of miR-145. **b** miR-145 promoted apoptosis of ovarian cancer cells, which was counteracted by overexpression of YTHDF2. **c** overexpression of YTHDF2 reversed the migration repressed by overexpression of miR-145. **d** The effect of overexpression of miR-145 on the global mRNA m6A levels was offset by overexpression of YTHDF2. **e** The expression of YTDHF2 at mRNA levels after overexpression of miR-145 and YTHDF2. **f** The expression of YTDHF2 at protein levels after overexpression of miR-145 and YTHDF2. All experiments were carried out in triplicate and the results were presented as means ± SE. *P < 0.05, **P < 0.001, t-test

The overexpression of miR-145 inhibited the proliferation, migration, and apoptosis of ovarian cancer cells, which was attenuated by the overexpression of YTHDF2 (Fig. 4a–c). Similarly, the effect of overexpression of miR-145 on global mRNA m6A levels was offset by the overexpression of YTHDF2 (Fig. 4d). The quantitative real-time polymerase chain reaction and Western blot analysis results showed that, after the overexpression of miR-145, the expression of YTHDF2 decreased, which was reversed by the overexpression of YTHDF2 (Fig. 4e and f). To conclude, the overexpression of YTHDF2 rescued the miR-145-induced reduction of proliferation and migration in EOC.

## Discussion

Ovarian cancer is a common malignant tumor of the female reproductive system, and its mortality rate ranks the first in gynecological tumors [1]. Besides the difficulty in early diagnosis, the poor prognosis of patients is also attributed to tumor recurrence and metastasis [2]. However, the specific mechanisms of EOC have not been fully elucidated. Recent studies have focused on the relationship between the methylation of m6A mRNA and tumor development. Increasing evidence shows that the methylation of m6A mRNA is closely related to the occurrence and development of tumors. The expression level of m6A-related protein is an important regulatory factor that directly determines the pathological process of tumor development [6]. However, the m6A modification in the regulation of EOC is still poorly understood. This study investigated how YTHDF2 and miR-145 modulated EOC progression through m6A modification.

Methylation of m6A is a dynamic and reversible process, involving writers, erasers, and readers [21]. YTHDF2, as a reader of m6A modification, belongs to the YTH domain family. It is closely related to the malignancy of HCC and can be adjusted by recognizing the m6A site. The downregulated miR-145 in patients with HCC can directly target the 3′-untranslated region of YTHDF2 mRNA to inhibit the expression of YTHDF2, which may be a new target for the treatment of HCC [13]. YTHDF2 is closely related to the malignant degree of HCC and can regulate mRNA degradation by recognizing the m6A site, leading to the enhancement of HCC cell proliferation [22–24]. However, the role of YTHDF2 in ovarian cancer has not been elucidated. This study found that the expression of YTHDF2 was significantly upregulated in EOC tissues compared with normal ovarian tissues, indicating the involvement of YTHDF2 in promoting ovarian cancer. In addition, this study demonstrated that YTHDF2 promoted proliferation and migration, inhibited apoptosis, and reduced global mRNA m6A levels of EOC cell lines. Taken together, the results confirmed that YTHDF2 could promote proliferation and migration by decreasing global m6A levels.

MiR-145 is a newly discovered miR with significantly downregulated expression in breast cancer, cervical cancer, glioma, colon cancer, esophageal cancer, and nonsmall cell lung cancer [25, 26]. Many recent studies have found that microRNA is closely related to ovarian cancer [27, 28]. Previous studies confirmed that miR-145 regulated different biological functions of ovarian cancer by targeting different target genes [17–20]. However, the expression pattern and the m6A-regulated role of miR-145 in ovarian cancer were still unclear. The present study found that the expression levels of miR-145 and YTHDF2 had an inverse correlation in ovarian cancer tissues and cells. A crucial crosstalk occurred between miR-145 and YTHDF2 via a double-negative feedback loop, and YTHDF2 was the direct target gene of miR-145. A correlation was found between the methylation modification of RNA m6A and miRNA. On the one hand, the miRNA-targeting site showed that m6A was enriched, and miRNA could positively regulate the activity of METTL3; on the other hand, the miRNA synthesis depended on m6A methylation modification [14, 15]. The findings confirmed that the primary microRNA was methylated by methylase METTL3 and recognized by the RNA-binding protein DGCR8. Drosha was recruited to cut the double-stranded RNA and produce the precursor miRNA (pre-miRNA) [14]. These results indicated that m6A was a marker for the post-transcriptional modification in miRNA biosynthesis. The present study demonstrated that YTHDF2 inhibited miR-145 expression. However, the molecular mechanism of YTHDF2 regulating miR-145 needs further exploration.

## Conclusions

In summary, this study concluded that YTHDF2, an miR-145-repressed protein, promoted the proliferation and migration of ovarian cancer cells. It was novel in revealing the regulatory mechanism of YTHDF2 in ovarian cancer and demonstrating the involvement of miR-145 and YTHDF2 in m6A modification and progression of EOC. The findings on YTHDF2 and miR-145 may provide potential therapeutic targets of EOC.

## Data Availability

The datasets during and/or analysed during the current study available from the corresponding author on reasonable request.
